# Cerebral Cavernous Malformation Bleeding Following Cerebrospinal Fluid Diversion Surgery: A Case Report and Literature Review

**DOI:** 10.7759/cureus.58689

**Published:** 2024-04-21

**Authors:** Raghad Salem, Othman T Almutairi, Mohammed Albrahim, Najeeb Alomar

**Affiliations:** 1 Department of Neurological Surgery, King Saud Medical City, Riyadh, SAU; 2 Adult Neurosurgery, King Fahad Medical City, Riyadh, SAU; 3 Department of Surgery, Maternity and Children Hospital, Ad-Dammam, SAU; 4 Neurosurgery, King Fahad Medical City, Riyadh, SAU

**Keywords:** vascular anomaly, stroke, csf diversion procedures, brainstem lesion, cerebra cavernous malformation

## Abstract

Cavernous malformations (CM) are rare intracerebral vascular lesions occurring in the brain, or less commonly in the spine, with an annual bleeding risk of up to 1.1%. These lesions can be occult or present to signs and symptoms based on location or, more frequently, are a result of hemorrhagic events. The most challenging aspect of managing these cases is weighing the risks and benefits of surgical treatment and intervening before the onset of a devastating hemorrhagic event. Here, we present the second case of CM haemorrhage following the cerebrospinal fluid (CSF) diversion procedure with a literature review of theories explaining this phenomenon.

We present a 37-year-old female who has a known case of brainstem cavernoma and underwent left sub-temporal resection with stable residual since 2011, then was managed conservatively due to patient preference till she had a deterioration in December 2021 manifested as confusion, diplopia, dysarthria, and significant left sided weakness leaving her wheelchair bound. CT showed supratentorial hydrocephalus with extensive periventricular transependymal edema and no clear haemorrhage. A ventriculoperitoneal (VP) shunt was inserted, with no intraoperative complications. A few hours post-VP shunt insertion, she experienced a worsening in her mental status, hemiparesis, and dysarthria. Subsequent imaging found evidence of acute haemorrhage in the location of the previously noted residual. She was managed by supportive care.

Causative factors of CM haemorrhage are poorly understood, and current data only suggest that prior haemorrhage and CM location could increase bleeding risk. Only one case of CM bleeding post-shunt insertion was reported; however, studies on other types of intracranial vascular lesions suggest that alterations in transmural pressure (including cerebrospinal fluid diversion procedures) can increase the risk of haemorrhage by changing the hemodynamic flow in these abnormally formed and weak vascular structures.

## Introduction

Cavernous malformations (CM) are low-flow vascular formations that consist of abnormally proliferating endothelium-lined leaky cavities or “caverns” containing blood of different ages [1]. CM is the third most common non-neoplastic cerebral vascular anomaly with a prevalence of 0.4% in the general population [2]. Sporadic cases of CM represent 80% of reported cases and the acquired cases represent the minority. The incidence of sporadic CM due to cranial radiation accounts for 3.9% in 10 years and 5% after 15 years post-radiation [3]. Familial cases account only for 20% of all cases and are inherited in autosomal dominant pattern [4,5]. It is known that familial CM presents with more aggressive behaviour and higher bleeding risk. In one study, 83% of cases with multiple CM who initially presented as sporadic cases were in fact familial [4]. CM can be found intracranially or in the spine. 

Despite recent research surge on CM, the aetiology is not fully understood. The majority of CM are asymptomatic and remain occult for a long period of time [5]. However, this is unlikely in the case of brainstem CM due to its critical location and can cause progressive deterioration in the neurologic baseline. Symptoms vary depending on location and can include seizure, headache, neurological defect, sensory disturbances, ataxia, weakness, vertigo, and decreased mental status. Frequently, Patients will present with different combinations of neurological signs and symptoms rather than a single complaint [5,6]. A new or worsened symptom can occur without bleeding and is often referred to as “non-haemorrhagic clinical event” [6]. The preferred imaging modality for CM is MRI with standard sequences and susceptibility-weighted imaging (SWI) [7]. They classically appear as heterogeneous “popcorn lesions” with blooming defects on SWI due to the various stages of bleeding and calcification occurring within the cavernoma. When managing CM cases, two points must be taken into consideration: managing the CM itself and alleviating the symptoms (i.e., headache, seizures, etc). Selecting the appropriate management modality for these lesions can be a dilemma. The outcome is favourable with surgical resection; however, stereotactic radiological surgery is currently trending, especially for deeply situated CM [1]. The most challenging factor in the management of CM is weighing the risk of haemorrhage and early surgical intervention. The only proven risk factors for CM bleeding include brainstem location and previous bleeding. Here, we present the second case of CM bleeding following CSF diversion [8] and discuss the effect of intracranial pressure (ICP) changes on the intramural cavernoma pressure changes relevant to CM bleeding.

## Case presentation

**Figure 1:**
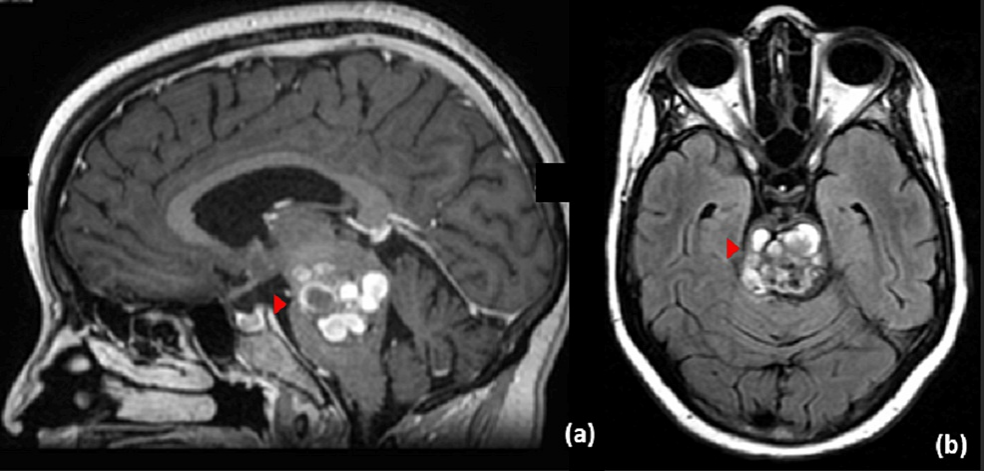
Pre-operative magnetic resonance imaging Pre-operative sagittal (a) and axial (b) cut of magnetic resonance imaging showing a mass lesion occupying the pontine isthmus with variable signal intensities measuring about 21 x 17 mm that is consistent with cavernous hemangioma causing local mass effect.

A 37-year-old female, following as a case of pontine CM, presented to the clinic with a new onset of right-sided hemiparesis, dysphagia, dysarthria, right facial palsy, right eye esotropia, and sensorineural deafness in the right ear. Magnetic Resonance Imaging (MRI) revealed a cavernoma occupying the pontine isthmus measuring 21 x 17 mm with variable intensities corresponding to different stages of bleeding (Figure 1).

**Figure 2:**
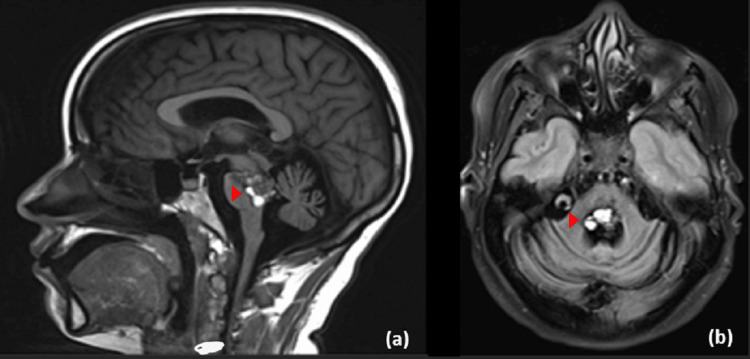
Post-operative magnetic resonance imaging Post-operative sagittal (a) and axial (b) cut of magnetic resonance imaging showing an interval increase in the size of the brainstem cavernoma that now measures 21 x 20 mm but no evidence of new hemorrrhage.

She underwent left sub-temporal resection in 2011 leaving a small residual in the pons measuring 19 x 17 mm (Figure 2).

**Figure 3:**
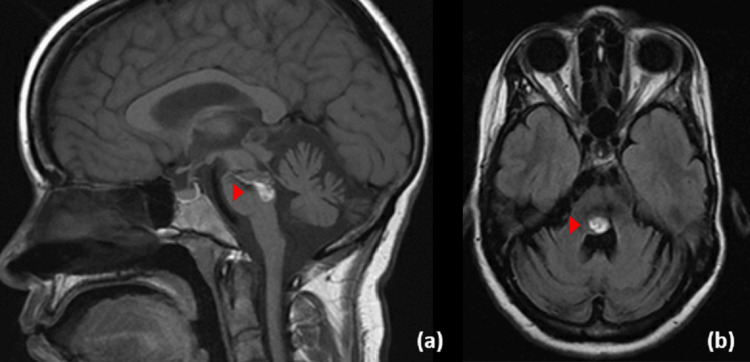
Follow-up contrast magnetic resonance imaging Sagittal (a) and axial (b) cut of magnetic resonance imaging showing a contrast-enhancing lesion with a newly appearing focal area of subacute bleeding noted at the site of the known Ponto-mesencephalic cavernoma with associated old blood degradation products.

On subsequent follow-up, she retained the same level of neurological function and mass size. In 2014, there was an interval increase in the residual mass that measures 21 x 16 x 23 mm without an acute well-established territorial infarction or intracranial haemorrhage (Figure 3). As the patient did not develop any new deficits and did not report any high intracranial pressure symptoms, she refused surgical intervention and she was followed in the clinic.

**Figure 4:**
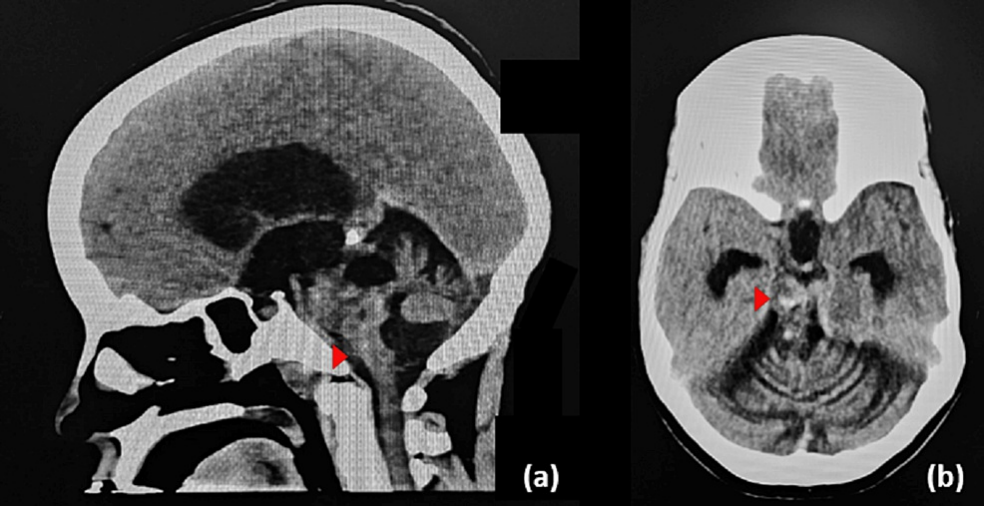
Follow-up plain computed tomography imaging The follow-up sagittal (a) and axial (b) cut of plain CT scan showing interval stability of previously noted pontine cavernous malformation.

**Figure 5:**
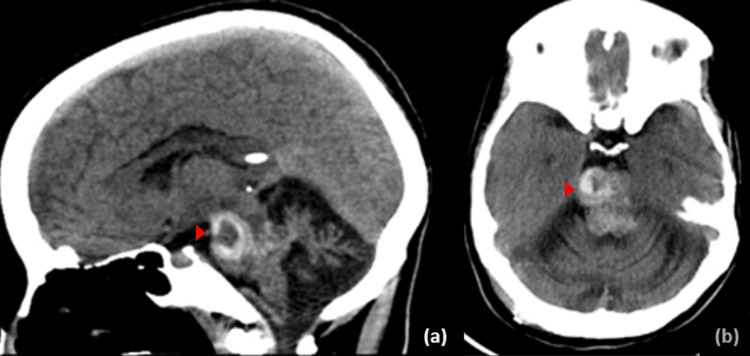
Urgent plain computed tomography imaging Sagittal (a) and axial (b) cuts of non-contrasted computed tomography scans showing an increase in the size of ventricles.

In December 2021, she presented with a 10-day history of worsening of right hemiplegia associated with facial numbness, slurred speech, right-sided ⅖ weakness, and left finger asymmetria. Radiological Correlation remained stable (Figure 4). The patient refused surgical intervention and was sent home. A few days later, she presented to the emergency department with loss of consciousness for 10 minutes secondary to a seizure confirmed by electrophysiological monitoring lasting for 10 minutes that occurred twice in the previous three days with overall worsening in her neurological baseline over two weeks. CT brain showed an interval increase in the size of the supratentorial ventricles with transependymal edema suggestive of communicating hydrocephalus with stability in the previously resected CM (Figure 5).

**Figure 6:**
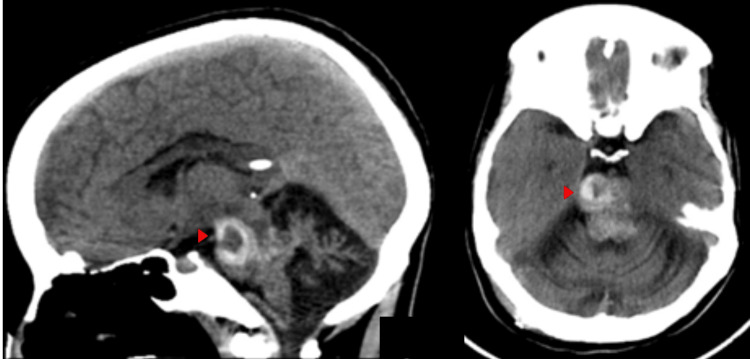
Urgent plain computed tomography imaging of the brain post shunt insertion Post shunt insertion sagittal (a) and axial (b) cut of non-contrast computed tomography scan showing lesional hyperdensity and regression in the size of the ventricles.

The patient was admitted for ventriculoperitoneal (VP)-shunt insertion. A medium-pressure shunt was utilised in this case, but the surgery was uneventful, and the patient regained full consciousness postoperatively. Immediate post-op MRI showed no intracranial haemorrhage or stroke and regression in the size of the ventricles. A few hours after shunt insertion, the patient had an acute deterioration in Glasgow Coma Scale (GCS) score of 10/15 (E4 V2 M4), National Institute of Health (NIH) Stroke scale was 28. Plain CT showed a collapsed supratentorial ventricular system with interval progression of the rounded hyper-density surrounding the cavernoma measuring 14 x 18 mm (Figure 6). The patient was admitted to the intensive care unit for three days in which she was managed conservatively and then tracheostomized. Three days later, she was then downgraded to a high dependency unit where she stayed for 21 days as she developed aspiration pneumonia that was treated. Upon discharge the patient GCS was 12/15, she had left sided hemiplegia and a motor power on the right upper limb was 3/5, hand grip was (4/5), right lower limb (1/5) with baseline cranial nerve deficits. The patient was sent to a long-term care facility for rehabilitation and recovery.

## Discussion

The risk of CM-related haemorrhage has been studied heavily in the literature. It is of high importance to differentiate between “Risk of bleeding” and “Risk of rebleeding”. In the literature, two established factors were found to increase the rate of haemorrhage: the occurrence in the brainstem and previous cavernoma-related bleeds. It was hypothesised that other contributing factors such as: size ≥10 mm, age <35 years, female gender, multiple CM, and presence of concurrent venous anomalies might increase the bleeding risk, but it was later rejected due to inconsistency [9]. Brainstem CM makes up 9-35% of intracranial CM [2]. The higher occurrence of CM in the midbrain compared to other locations in the brainstem accounts for a higher rate of perioperative morbidity and mortality [6]. Recurrent bleeding in brainstem CM is common with a reported risk reaching up to 21% per patient per year [9]. One study even reported that 56% of patients experienced rebleeding before surgery and 22% had more than two bleeding episodes [5]. It is known that the morbidity of re-bleeding is higher than the first bleeding event [5]. The relation between changes in ICP and bleeding risk is well established in other vascular malformations such as cerebral and aortic aneurysm and arteriovenous malformation [8]; however, only one case report exists that describes this phenomenon in CM [8]. Similarly, in our case, a patient with a previously bled left atrial CM with a presentation of hydrocephalus that was treated with programmable Anti-Siphon ventriculoperitoneal shunt in which CM-related haemorrhage was stopped after multiple trials of valve rate adjustments [8].

The observed CM-related bleeding after CSF diversion can be hypothetically explained by the following three factors: fragility of these abnormal vessels, venous dysfunction, and neurohormonal dysfunction. CM consists of abnormally thin-walled dilated vessels with abnormal endothelial cell junctions. CM lacks subendothelial support and basal lamina deposition of disorganised collagen bundles [10]. It’s suggestive that these structurally incompetent vessels are sensitive to trans-mural pressure alteration due to the transmitted pressure gradient across the dysfunctional blood-brain barrier (BBB), which justifies its inherited tendency to bleed after sudden intracranial pressure changes. Secondly, recent studies postulated that in CM an increase in venous flow due to non-hemorrhagic venous dilatation with a decrease in venous outflow represents reduced structural venous integrity yet to be proved to be related to haemorrhagic events [11]. In our case, the co-existence of increased ICP and its related increase in venous pressure have resulted in a transmural accentuation of pressure changes with CSF diversion and the hypothesis of blood extravasation. It’s known that in pregnant ladies, the natural history of CM-related haemorrhagic events is not different from that of non-pregnant ladies [12], knowing that in pregnancy and peripartum states, there are fluctuations in venous circulation from insufficiency to postpartum transient hypertension [12]. Interestingly, in pregnancy and peripartum states, there are fluctuations in venous circulation from insufficiency to postpartum transient venous hypertension [12]. This observation questions the effect of venous pressure irregularities on the risk of haemorrhage especially when venous changes are gradual as in pregnancy, but sudden changes of the venous regulations might be less adapted hypothetically.

Lastly, it is known that an increase in the ICP produces a linear incremental correlation with sympathetic activity [13]. The internal Jugular Vein (IJV) autonomic regulation has been postulated to affect the venous outflow by the adrenergic activation of IJV receptors increasing tone and related luminal pressure with fluctuation in switching on/off the receptor can result in transient IJV vasospasms. The concomitance of venous alteration with CM can produce venous endothelial micro-tears and extravasation of blood [11].

Due to the patient's decision to abstain from surgery, she was managed conservatively with frequent visits in the outpatient setting. It is very difficult to determine whether a more favourable outcome was possible for such a case as brainstem CM are known to have an unfavourable outcome even with neurosurgical excision due to their critical location and higher tendency to bleed [14]. The efficiency of newly emerging treatment modalities such as stereotactic radiosurgery is yet to be determined [14]. The possibility of medical management (i.e., propranolol, NSAIDS) in reducing the risk of bleeding in CM has been the topic of interest in many clinical trials [14].

## Conclusions

This case report explores new concepts when managing patients with CM with high ICP. The bleeding likelihood of CM is mainly dependent on location and previous hemorrhagic event; However, newly emerging evidence suggests that rapid change in transmural pressure following CSF diversion procedures may be an additional factor. Although it is a well acknowledged phenomenon in other cranial vascular lesions, it is uncommon in CM and is only found in one other case report so the causality between these two factors can be difficult to prove.
